# The Distinct Roles of Sialyltransferases in Cancer Biology and Onco-Immunology

**DOI:** 10.3389/fimmu.2021.799861

**Published:** 2021-12-17

**Authors:** Marjolaine Hugonnet, Pushpita Singh, Quentin Haas, Stephan von Gunten

**Affiliations:** ^1^ Institute of Pharmacology, University of Bern, Bern, Switzerland; ^2^ Bern Center for Precision Medicine (BCPM), University of Bern, Bern, Switzerland

**Keywords:** tumor glycosylation, sialyltransferases, sialic acid, cancer, tumor immunology

## Abstract

Aberrant glycosylation is a key feature of malignant transformation. Hypersialylation, the enhanced expression of sialic acid-terminated glycoconjugates on the cell surface, has been linked to immune evasion and metastatic spread, eventually by interaction with sialoglycan-binding lectins, including Siglecs and selectins. The biosynthesis of tumor-associated sialoglycans involves sialyltransferases, which are differentially expressed in cancer cells. In this review article, we provide an overview of the twenty human sialyltransferases and their roles in cancer biology and immunity. A better understanding of the individual contribution of select sialyltransferases to the tumor sialome may lead to more personalized strategies for the treatment of cancer.

## Introduction

Cancer remains one of the leading cause of death worldwide (1). During their development, cancer cells undergo important genetic and structural modifications (2). A well-known feature of malignant transformation is aberrant glycosylation (3, 4). Altered tumor glycosylation was initially described in the mid-twentieth century (5–7), and has since been studied in-depth with regard to its role in tumor progression. Tumor-specific glycosylation has been linked to many processes involved in oncogenesis, such as tumor growth and progression, invasion, metastasis, angiogenesis, chemoresistance and tumor immunity (3, 4, 8–12).

Commonly found glycosylation changes in cancer cells include hypersialylation, incomplete synthesis, truncation of O- and N-glycans, altered branching, and even xenoglycosylation (3, 13). Hypersialylation, referring to the increased density of sialic acid-containing glycans (sialoglycans), is one of the most common features of altered tumor glycosylation (3). Overexpressed sialoglycans include sialylated derivatives of Lewis antigens (sialyl-Lewis X [sLeX]), sialyl-Lewis A [sLeA]), which as ligands of selectins are long known to promote tumor metastasis (3, 14). Accumulating evidence suggests that distinct sialoglycans act as glycoimmune checkpoints that suppress anti-tumor immune reactivity by engagement of immunoregulatory Siglec receptors on myeloid and lymphoid immune cells (12, 15–17). Indeed, ligands of Siglecs are broadly expressed on primary human cancer cells and cell lines of different origin (18).

In humans, twenty different sialyltransferases (SiaTs) are involved in the biosynthesis of glycans and each exhibits distinct characteristics and preferences such as for substrates and glycosidic linkages. The expression levels of individual SiaTs varies significantly between different types of tumors (19), but also within tumors of the same origin (20). While the overexpression of certain sialyltransferases in cancer is associated with tumor hypersialylation and adverse outcome, such positive correlation is not found for all sialyltransferases and may also depend on the type of tumor (see below). Given the significance of distinct sialylation patterns for cancer biology and immunity, in this review article we provide an overview on expression and roles of individual sialyltransferases in cancer.

## Sialic Acids and Sialyltransferases

Sialic acids (neuraminic acids) are nine-carbon (C1-9) monosaccharides most commonly found at a terminal position on the outer end of glycoconjugates on many glycoproteins and glycolipids synthesized by living cells (21). Their prominent position on the cell surface glycans of mammalian cells keeps them at the forefront of cellular processes in health, but also in cancer biology and immunity (22–25).

**Figure 1:**
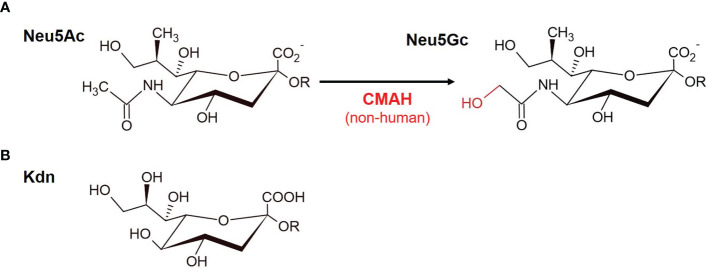
Sialic acids. Sialic acids are nine-carbon monosaccharides. **(A)** The two main mammalian sialic acids N-acetyl neuraminic acid (Neu5Ac) and N-glycolylneuraminic acid (Neu5Gc) are shown. Neu5Gc is derived from Neu5Ac and differs by one oxygen atom in the N-glycolyl group, which is added by the enzyme cytidine monophosphate N-acetylneuraminic acid hydroxylase (CMAH) in the cytosol. Humans have an inactivating mutation of the CMAH gene and therefore they lack this enzymatic activity. **(B)** Kdn (2-keto-3-deoxy-D-glycero-D-galacto-nononic acid), which is more common among lower vertebrates and bacteria (see text).

The most prevalent sialic acids in mammals comprise N-acetylneuraminic acid (Neu5Ac) and N-glycolylneuraminic acid (Neu5Gc) monosaccharides (**Figure 1A**). 2-keto-3-deoxy-D-glycero-D-galacto-nononic acid (Kdn) sialic acids are more widespread in lower vertebrates (26) (**Figure 1B**). When one or more hydroxyl groups of Neu5Ac, Neu5Gc or deaminated neuraminic acid (Kdn) are substituted with acetyl, methyl or sulfate residues, more than 50 derivatives with a high diversity are formed (21, 27). As opposed to most mammals, humans do not naturally express Neu5Gc due to the deletion of the *CMAH* (Cytidine monophospho-N-acetylneuraminic acid hydroxylase) gene, which is responsible for the conversion of Neu5Ac into Neu5Gc (28) (**Figure 1A**). It is thought that the deletion of this gene could have provided selective advantages during human evolution and eventually played a role in brain development and running endurance in humans (29, 30). Remarkably, Neu5Gc is often expressed in glycoconjugates of human tumors (13, 31, 32). Due to altered metabolic pathways tumor cells are able to incorporate non-human Neu5Gc (3, 13, 33, 34), which humans can retrieve from foods such as red meat (35, 36).

**Figure 2:**
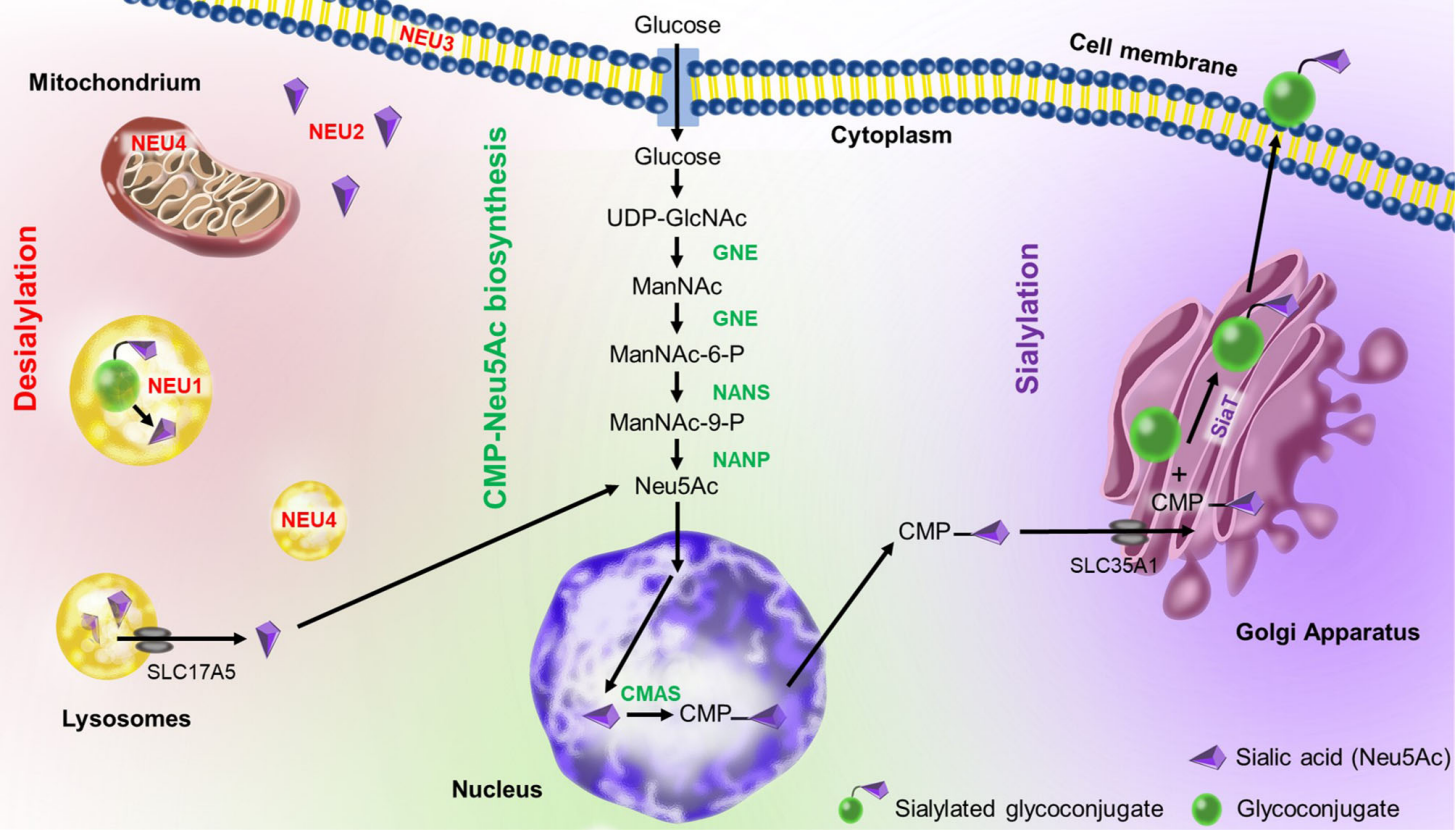
Sialic acid metabolism in humans. CMP-Neu5Ac mostly occurs in the cytoplasm except of the CMP-sialic acid synthase (CMAS)-mediated reaction which takes place in the nucleus. UDP-GlcNAc-2 epimerase (GNE) synthesizes N-acetylmannosamine (ManNAc) in two steps. Then, Neu5Ac synthase (NANS) generates ManNAc-9-P, which is then dephosphorylated by Neu5Ac-P-phosphatase (NANP) to generate free sialic acid in the cytoplasm. The free sialic acid can enter the nucleus to be linked to CMP (cytidine-5’-monophosphate). The CMP-Neu5Ac is transferred to the Golgi apparatus *via* SLC35A1 transporter (solute carrier family 35 member A1), where it is used as a substrate for sialylation by different sialyltransferases (SiaT). Sialylated glycoconjugates are then exported to the cellular membrane or secreted. They can also be broken down by various neuraminidases (NEU1-4) present in different cellular localizations. The released sialic acid can reenter the biosynthesis pathway. Illustration by Aldona von Gunten.

The sialic acid metabolism involves enzymes that catalyze the biosynthesis and transfer of sialic acid to a glycoconjugate, as well as the removal and degradation of sialic acid (37) (**Figure 2**). Sialic acid biosynthesis starts with UDP-GlcNAc (uridine diphosphate N-acetylglucosamine) produced *via* the hexosamine pathway, which is converted to ManNAC-6-P (N-Acetyl-mannosamine 6-phosphate) by UDP-GlcNAc 2-epimerase/ManNAc-6 (GNE) in a two-step process (38). Then, Neu5Ac synthase (NANS) generates 9-phosphorylated forms of sialic acid (Neu5Ac-9-P), which is then dephosphorylated by Neu5Ac-P-phosphatase (NANP) to generate free sialic acid (Neu5Ac) in the cytoplasm (39). Next, cytosolic Neu5Ac enters the nucleus and is activated by coupling cytidine monophosphate (CMP) *via* the action of cytosine 5’-monophosphate N-acetylneuraminic acid synthetase (CMAS) to produce CMP-Neu5Ac (40). CMP-Neu5Ac is used by sialyltransferases in the Golgi apparatus for sialylation of glycoconjugates. Finally, sialylated glycoproteins and glycolipids are exported to the cell membrane or secreted.

On the other hand, sialic acid can also be released by neuraminidase (also called sialidase) from sialylated glycoconjugates (40). There are 4 mammalian neuraminidases with different cellular localizations: the lysosomal neuraminidase NEU1 (41), the cytosolic neuraminidase NEU2 (42), the plasma membrane-associated neuraminidase NEU3 (43) and the lysosomal or mitochondrial membrane-associated neuraminidase NEU4 (44). The released sialic acids can be reutilized in the biosynthesis pathway (40). Hypersialylation, as occurring in malignancy, is closely associated to an imbalance between sialic acid biosynthesis and desialylation (45).

**Figure 3:**
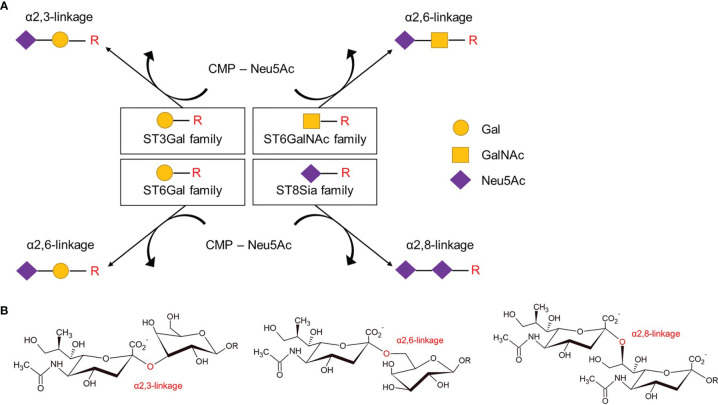
Members of the four families of sialyltransferases catalyze different glycosidic linkages. **(A)** The four families of sialyltransferases as categorized according to restricted glycosidic linkage and acceptor specificity. Indicated are the transfer of activated CMP-Neu5Ac onto Gal, GalNAc or Neu5Ac moieties of carbohydrate chains (-R), such as on glycoproteins or glycolipids. **(B)** Examples of glycosidic α2,3, α2,6, and α2–8 -linkages involving the hydroxyl group at carbon atom 2 of Neu5Ac sialic acid with galactose (left, middle) or another sialic acid (right). CMP, cytidine monophosphate; Neu5Ac, N-acetylneuraminic acid; Gal, galactose; GalNAc, N-acetylgalactosamine.

Human SiaTs comprise a set of 20 glycosyltransferases which all use cytidine monophosphate N-acetylneuraminic acid (CMP-Neu5Ac) as an activated sugar donor for the transfer of sialic acids to the terminal glycosyl group of glycoproteins and glycolipids as acceptor molecules (46). SiaTs catalyze the formation of different glycosidic linkages, α2,3-, α2,6-, or α2,8-linkage, and also vary in their acceptor specificities. Accordingly, SiaTs can be grouped into four different families: ST3Gal, ST6Gal, ST6GalNAc, and the ST8Sia (**Figure 3**). Even though SiaTs share the same sugar donors, they present specific substrate specificity, although with some degree of redundancies. Indeed, enzymatic analysis conducted *in vitro* with recombinant enzymes revealed that one linkage can be synthesized by multiple enzymes (47, 48). SiaTs share conserved sialylmotifs, including ‘L’- (for long), ‘S’- (for short), ‘III’ (for being third position in sequence), and ‘VS’- (for very small) motifs (49). The L-motif is thought to mediate the binding of the donor substrate, the III- and VS-motifs bind the acceptor substrate, and the S-sialylmotif contributes to both binding of donor and acceptor substrates (49). A disulfide bond between the L- and S-motifs bring all sialylmotifs closer together to facilitate interactions with substrates (49).

SiaTs have been shown to be primarily restricted to medial- and trans-cisternae of the Golgi apparatus, with some being present in the trans-Golgi network (50), but some SiaTs are also expressed as post-Golgi and secreted enzymes (51, 52), and SiaT activity was also reported to occur at the cell surface of monocyte-derived dendritic cells (53). Their expression pattern among tissues is diverse, but some SiaTs are preferentially expressed at distinct sites. For specific protein expression of SiaTs the Human Protein Atlas (54) can be consulted (proteinatlas.org).

Increased activity or expression of SiaTs leads to the hypersialylation of cell surfaces which is one of the most common glycosylation changes that occurs in tumors; it entails the enhanced expression of sialic acid-terminated glycoconjugates (3). Many studies show elevated levels of SiaTs in the plasma of cancer patients (55–58). The relative diversity and complexity of sialylation patterns in tumors represents a promising area of research, knowing that each SiaT is involved in the synthesis of various structures, therefore, broadly impacting cancer development in various ways, which will be discussed in the following sections.

## ST3Gal Family

**Figure 4:**
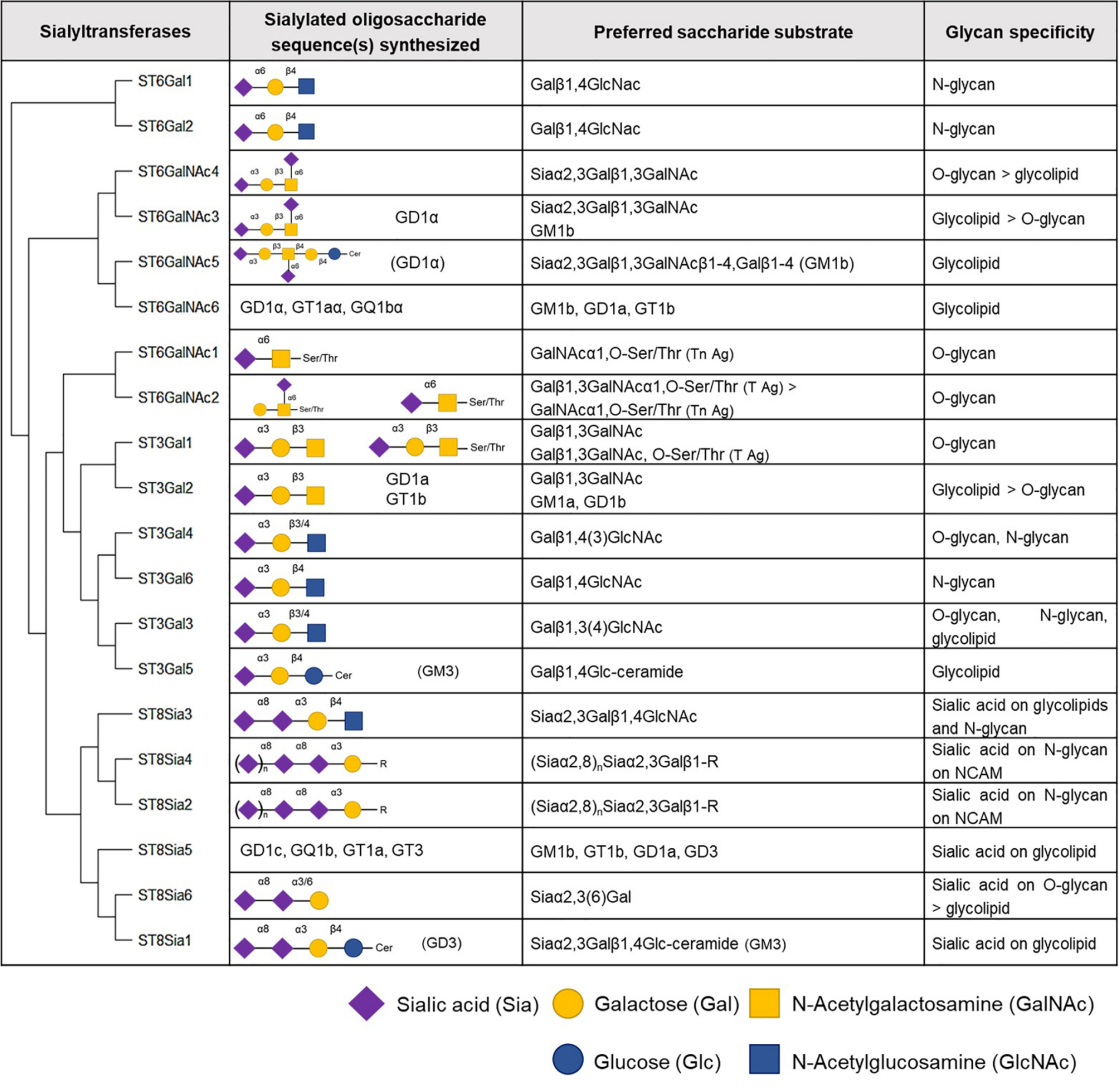
Human sialyltransferases. The twenty human sialyltransferases listed according to their homology (60). Select generated oligosaccharides, preferred substrates and glycan specificities of individual sialyltransferases are shown.

Six β-galactoside α2,3-sialyltransferases belong to the ST3Gal family in humans and these enzymes transfer sialic acid residue in an α2,3-linkage to terminal galactose (Gal) residues present on glycolipids or glycoproteins (59, 60). Members of this family are involved in the synthesis of gangliosides (ST3Gal2 and 5), and the tumor-associated sialyl-T (ST) (ST3Gal1) and sialyl-Lewis (ST3Gal3, 4, and 6) antigens (**Figure 4**).

### ST3Gal1

ST3Gal1 is known as the major human SiaT to synthesize sialyl-T (ST) antigen from the T antigen Galβ1-3GalNAc. While the T and ST antigens are found on normal *O*-glycans such as in hematopoietic cells (4), ST3Gal1 overexpression is found in different types of malignancies (61–65), and has been linked to poor prognosis (65, 66). MUC1-ST, a glycoform of the mucin MUC1 carrying the ST antigen found in breast cancer patient serum (67), through Siglec-9 engagement, triggers the differentiation of a unique tumor-associated macrophage (TAM) subtype that has been associated with poor prognosis in breast cancer (68). Recently, Rodriguez et al. identified ST3Gal1 as a main contributor to the synthesis of Siglec-7 ligands in pancreatic cancer cells, which by engagement of the sialic acid-Siglec axis may shift TAM differentiation towards a more suppressive phenotype (69). Overexpression of ST3Gal1 has been shown to promote tumor cell migration and metastasis (65, 70–72), which may involve epidermal growth factor receptor (EGFR) signaling (72), or receptor tyrosine kinase AXL dimerization/activation (71). Moreover, ST3Gal1 seems to also play a role in TGF-β1-induced epithelial-mesenchymal transition (EMT) in ovarian cancer cells (70). ST3Gal1 is also enrolled in promoting resistance to anti-cancer effects of agents, such as of adriamycin directed against chronic myeloid leukemia (CML) cell lines (73), paclitaxel against ovarian cancer cells (70), and tamoxifen and/or vandetanib against breast cancer cells (66). The exact mechanisms of ST3Gal1-mediated resistance to chemotherapeutic drugs remain to be deciphered.

### ST3Gal2

*In vivo* genetic experiments showed that ST3Gal2 is a key enzyme mediating α2,3 sialylation of gangliosides in the brain of mice, in particular of GD1a and GT1b, eventually with support of ST3Gal3 (74). *ST3GAL2* mRNA expression was found to be associated with advanced stage and poor clinical outcome in cancer (75, 76). Increased mRNA expression of *ST3GAL2*, as well as *ST3GAL5* and *ST8SIA1*, was also observed in breast cancer stem cells which is eventually linked to increased expression of gangliosides in these cells (77). ST3Gal2 is a rate-limiting enzyme for SSEA-4 (sialyl-glycolipid stage-specific embryonic antigen 4) synthesis (78), which was shown to be limited in normal tissues but highly expressed in glioblastoma cells (79) and has been associated with epithelial-mesenchymal transition (EMT) (76), loss of cell-cell interactions and adaptation of a migratory phenotype (80). Furthermore, a positive correlation between SSEA4 and chemoresistance was reported (76). Notably, gangliosides are differentially recognized by the immunoregulatory receptors Siglec-7 and -9 receptors (81, 82).

### ST3Gal3

ST3Gal3 is involved in the synthesis of sLeA (also known as carbohydrate antigen 19-9 [CA19-9]) and sLeX, which are expressed in different types of cancer (83–87), and have been linked to cancer progression and poor prognosis (88), eventually by selectin-mediated invasion and metastasis of tumor cells (14, 89). Indeed, the expression of *ST3GAL3* in breast cancer was found to be associated with poor prognosis (90). ST3Gal3 has also been associated with paclitaxel and cisplatin resistance in ovarian cancer cells (91, 92).

### ST3Gal4

ST3Gal4 is involved in the biosynthesis of the tumor-associated antigen sLeX (89, 93). *ST3GAL4* expression correlates with enhanced metastatic potential and poor prognosis in some types of cancer, including pancreatic and gastric cancer (94, 95), which may involve selectin-dependent adhesion through sLeX (87). Recently, ST3Gal4 was found to be responsible for the generation of ligands for the immunoregulatory receptor Siglec-9 in pancreatic cancer cell lines (69), and Siglec-7 and -9 ligand in HEK293 cells (96), indicating its potential role in the generation of glyco-immune checkpoints. However, overexpression of *ST3GAL4* appears not to be a universal feature of malignancy as downregulation of the enzyme or specific variants has been found for instance in premalignant and malignant cervical tissues (97) and renal cell carcinoma (98). Tissue-specific transcriptional regulation involving alternative splicing and promoter utilization has been described for alpha2,3-sialyltransferases (99), and may explain the differential expression in various types of malignancies.

### ST3Gal5/GM3 Synthase

ST3Gal5 initiates the biosynthesis of many downstream gangliosides (100), and is also known by the name “GM3 synthase”. GM3, the simplest ganglioside, is involved in various processes such as transmembrane signaling through the regulation of growth receptor activities and in integrin-mediated cell adhesion and motility (101, 102). Furthermore, GM3 has been shown to be recognized by inhibitory Siglec-9 (103). However, ST3Gal5 also mediates the synthesis of GM4 (104). In a breast cancer model, GM3 synthase knockout mice exhibited enhanced tumor growth and angiogenesis (105). In bladder cancer, the downregulation of ST3Gal5 was associated with reduced patient survival (106). Such experimental evidence suggests a beneficial role of GM3 synthase and certain products, such as distinct a-, b- and c-series gangliosides eventually, in at least some tumors. However, given that GM3 synthase acts at an early stage of ganglioside biosynthesis, it remains unclear which ganglioside products and derivatives are effective in such experimental models and differences may exist among different types of tumors.

### ST3Gal6

Like ST3Gal3 and ST3Gal4, ST3Gal6 mediates the sialylation of LeX antigen (83). The resulting sLeX antigen interacts with selectins, such as during the initial tethering before extravasation of cells (107). Indeed, ST3Gal6 was shown to have a crucial role in the generation of selectin ligands in mice (108). High expression of ST3Gal6 in multiple myeloma (MM) patients is associated with poor prognosis (109). Knockdown of *ST3GAL6* resulted in a reduced surface expression of α-2,3-linked sialic acid and sLeX on MM cell lines and also reduced the homing and engraftment of malignant cells to the bone marrow niche *in vivo* (109). Furthermore, mice injected with *ST3GAL6* knockdown MM cells demonstrated a decreased tumor burden and prolonged survival. Higher expression of Lewis antigens in neuroblastoma MYCN-amplified cell lines and patient samples could be a consequence of the overexpression of SiaTs, including ST3Gal3/4/6, compared to MYCN-non-amplified counterparts (110). Furthermore, high-grade glioma cell lines exhibit higher expression of terminal sLeX and of the SiaTs ST3Gal3/4/6 compared to low-grade glioma cells (111). ST3Gal6 is also upregulated in human hepatocellular carcinoma (HCC) tissues, and correlates with cell proliferation, migration and invasion ability in HCC cell lines (112). Similar observations were made in urinary bladder cancer with a positive correlation between increased *ST3GAL6* expression and tumor stage, grade as well as poor outcome (113).

## ST6Gal Family

ST6Gals preferentially link sialic acids in an α2-6 linkage to galactose residues of Galβ1-4GlcNAc-R on N-glycans (59, 60). This family contains two enzymes ST6Gal1 and ST6Gal2, and is thus the smallest SiaT family.

### ST6Gal1

ST6Gal1 is the main sialyltransferase contributing to the addition of α-2,6-linked sialic acid to Galβ4GlcNAc chains, usually present in N-linked chains (59). ST6Gal1 is frequently overexpressed in many solid tumors, such as pancreatic, gastric, cervical, ovarian, brain and colorectal cancers and cancer cell lines (114–120). Indeed, this enzyme has been extensively investigated in regard to cancer research [for a review see (121)]. High expression of *ST6GAL1* in cancer correlates with worse tumor grade (90, 122), advanced stage of disease (120), and poor prognosis (119, 120, 122). While a greater number of experimental studies support an oncogenic role of ST6Gal1 (discussed below), few reports propose an inverse role of this enzyme based on evidence from select *in vitro* and *in vivo* experimental models (123–125). Interestingly, while ST6Gal1 mRNA expression was found to be increased in papillary non-invasive bladder tumors, expression of this enzyme was found to be decreased in muscle-invasive bladder cancer due to epigenetic inactivation of *ST6GAL1* by promoter methylation (126).

Interestingly, ST6Gal1 was shown to protect tumor cells from hypoxic stress, eventually by enhancing the expression of hypoxia-inducible factor-1α (HIF-1α) (127). ST6Gal1 activity has been shown to promote EMT in cell lines of different histological origin (128–130), eventually involving E-cadherin transcription and turnover, as well as PI3K/Akt signaling (128). Silencing of ST6Gal1 in prostate cancer cell lines resulted in decreased expression of components of the PI3K/Akt and β-catenin signaling pathways, resulting in reduced proliferation, migration and invasion (122). Furthermore, ST6Gal1 expression is associated with nonmalignant stem and progenitor cells, but also with stemness in cancer and may drive cancer stem cell (CSC)-like characteristics (131–136). Furthermore, high expression of *ST6GAL1* in CSCs could eventually promote chemo-resistance (137). Indeed, ST6Gal1 has been linked to resistance to a number of agents including gemcitabine (138), cisplatin (139), trastuzumab (140, 141) or gefitinib (142), latter of which appears to involve sialylation and activation of EGFR (142).

Several investigators observed that α2-6 sialylation by ST6Gal1 activity may protect cells from cell death, and eventually block homeostatic epithelial cell apoptosis in cancer (133). ST6Gal1-mediated sialylation prevents apoptosis induced by tumor necrosis factor receptor 1 (TNFR1) (143), eventually by restraining the receptor on the cell surface (144). Similarily, α2-6 sialylation of the death receptor FAS by ST6Gal1 prevents receptor activation by blocking its internalization and the subsequent formation of death-inducing signaling complex and activation of apoptotic caspase-dependent signaling pathways (145). Furthermore, sialylation of β1 integrins by ST6Gal1 conferred protection against galectin-3-induced apoptosis in a cancer cell line (146).

Recently, using gene engineered HEK293 cells, ST6Gal1 was found to be partially responsible for the generation of ligands for the immunoregulatory receptor Siglec-7 (96), indicating its potential role in the generation of glyco-immune checkpoints.

### ST6Gal2

ST6GAL2 is predominantly expressed in the adult brain and fetal tissues, and to a lesser extent in the thyroid gland, small intestine, colon, and testis (147, 148). While relatively few studies have investigated the expression and role of ST6GAL2 in tumors, overexpression of this enzyme was found in select types of cancer, including breast cancer (149) and follicular thyroid carcinoma (FTC) (150). In breast cancer ST6GAL2 expression associated with poor prognosis for patients (149). Moreover, silencing of *ST6GAL2* in breast cancer cells resulted in reduced xenograft tumor growth *in vivo* (149). Furthermore, this study revealed that *ST6GAL2* silenced cell lines exhibited reduced adhesion and invasion properties *in vitro*, with downregulation of several focal adhesion molecules (ICAM-1, VCAM-1) and metastasis pathways proteins (MMP2, CXCR4). Similarly, silencing of *ST6GAL2* in FTC reduced tumor growth in an *in vivo* model (150). Findings from this study suggest that the overexpression of *ST6GAL2* leads to the suppression of the Hippo signaling pathway, a tumor suppressor pathway that regulates cellular differentiation and proliferation by inhibiting YAP and TAZ transcription co-activators (151–153).

## ST6GalNAc Family

The six SiaTs of the ST6GalNAc family catalyze the glycosidic linkage of sialic acids to N-galactosamine (GalNAc) residues found on *O*-glycosylated proteins or glycolipids in an α2-6 linkage.

### ST6GalNAc1

ST6GalNAc1 catalyzes the generation of sialyl-Tn (sTn) antigen from Tn antigen (154). sTn is a well-known tumor-associated carbohydrate antigen (TACA) overexpressed in multiple cancers (155–157), and has been linked to poor prognosis (158–160). Expression of the biosynthetic enzyme ST6GalNAc1 has also been directly associated with poor prognosis (161). Indeed, overexpression of ST6GalNAc1 in gastric, breast, prostate and ovarian cancer cell lines and tissues has shown to induce the expression of the sTn antigen (155, 157, 161–166). The expression of ST6GALNAC1 can also be induced by cytokines, such as IL-13 and CCL17 secreted by M2 macrophages co-cultured with colon cancer cells, which may result in higher expression of sTn antigen including on MUC1 (167).

Downregulation of *ST6GALNAC1 via* hyper-methylation and loss of heterozygosity (LOH) was observed in esophageal carcinoma in tylosis, an inherited epithelial disorder (168). Interestingly, in prostate cancer a splice variant of ST6GalNAc1 is induced by androgens, which consists of a shorter isoform that exhibits sialyltransferase activity yet with slightly different properties (157).

In experimental models, overexpression of ST6GalNac1 reduced cell-cell aggregation and increased extracellular matrix (ECM) adhesion, migration and invasion *in vitro* (163, 166), and promoted tumor growth and metastasis *in vivo* (163, 164) (165). Furthermore, ST6GalNAc1 activity might foster cancer cell stemness, as expression of CSC markers and tumor sphere formation capability were increased in ST6GalNAc1 overexpressing colorectal or ovarian cancer cell lines (161, 164). Stemness through the generation of sTn seems to involve Akt pathway signaling (161, 164), eventually in cooperation with Galectin-3 (161).

The immunoreceptor Siglec-15 was shown to recognize sTn antigen (169, 170), and to depend on ST6GalNac1-mediated biosynthesis (170). Engagement of Siglec-15 by binding to tumor-associated sTn antigen resulted in enhanced TGF-β secretion from monocytes/macrophages following DAP12-Syk signaling (171). Notably, a recent study showed that macrophage-associated Siglec-15 suppressed T cell responses *in vitro* and *in vivo*, eventually establishing a mechanism for immune evasion in the TME (172).

### ST6GalNAc2

ST6GalNac2 synthesizes sialyl-6-T antigen from T antigen, and to a lesser extent it sialylates the Tn antigen (154, 166). High transcriptional expression of *ST6GALNAC2* correlated with poor prognosis in colorectal cancer (173), and was found to be associated with higher histological tumor grade, lymph node metastasis, and advanced clinical stage in FTC (174). ST6GalNAc2 has been proposed to enhance invasive properties of cancer cell lines *via* PI3K/Akt pathway signaling (174, 175). However, the role of ST6GalNac2 in cancer appears not to be unequivocally detrimental as Murugaesu and colleagues identified ST6GalNAc2 as a novel metastasis suppressor in mouse and human breast cancer models (176). Indeed, high levels of ST6GALNAC2 expression correlated with increased survival in patients with breast cancer (176). The authors showed that silencing of *ST6GALNAC2* modified the cell surface O-glycome resulting in an increase in unmodified T antigen/core 1 antigen and a reduction in the disialyl core 1 antigen. Such altered glycosylation facilitated the binding of the soluble lectin galectin-3 and resulted in increased tumor cell aggregation, pulmonary tumor cell retention and metastatic burden *in vitro* or *in vivo*.

### ST6GalNAc3

ST6GalNAc3 uses α2,3-sialylated ganglioside GM1b as a substrate to synthesize the ganglioside GD1α. In healthy individuals, this enzyme is highly expressed in brain and kidney (177). Aberrant promoter hypermethylation of *ST6GALNAC3* was found in prostate cancer tissue samples (178), but it remains to be shown whether transcriptional silencing of this gene influences the development or progression of prostate cancer. However, ST6GalNAc3 seems to promote the proliferation of A549 non-small cell lung cancer cells through enhanced expression of transferrin receptor protein 1 (TFR1) (179), which is important for cell proliferation and survival (180).

### ST6GalNAc4

ST6GalNAc4 mediates the synthesis of disialyl-T antigen from sialyl-T antigen (O-glycan), and also generates the disialyl-lactotetraosyl-ceramide GD1α from sialyl-lactotetraosyl-ceramide GM1b (gangliosides) yet to a lesser degree than ST6GalNAc3 (181, 182). Upregulation of ST6GalNAc4 and downregulation of the core 2 N-acetylglucosaminyltransferase C2GnT2 (*Gcnt3*) were shown to be key in conferring tumor cell glycosylation changes that contribute to metastatic activity in a primary lung cancer model, eventually by preserving presentation of the T-antigen and adherence to galectin 3 (183). In another study, higher expression of *ST6GALNAC4* was observed in FTC tissues compared to transitional tissues and silencing of this enzyme led to decreased invasive ability *in vitro* and *in vivo* (184).

### ST6GalNAc5/GD1α Synthase

ST6GalNAc5 transfers a sialic acid residue onto GM1b to form GD1α (185) and this enzyme is also referred to as GD1α synthase (186). Indeed, transfection of the human ST6GalNAc5 cDNA into a breast cancer cell line resulted in the expression of GD1α (187). A study investigating germline single-nucleotide polymorphisms indicates that specific SNPs of *ST6GALNAC5* determine susceptibility for colorectal brain metastasis and overall survival (188). Silencing of *ST6GALNAC5* in breast cancer cells led to decreased metastasis in a murine model *in vivo*, and in an *in vitro* model using human umbilical vein endothelial cells (HUVEC) silenced cells exhibited reduced blood brain barrier (BBB) transmigration activity (189). As opposed, a more recent study showed that ST6GalNac5 overexpression in breast cancer cells leads to a decreased adhesion and no change in transmigration compared to controls in a human BBB model using CD34+ hematopoietic stem cell derived endothelial cells co-cultivated with brain pericytes (190),. The authors of this study suggested that differences in the used BBB models may account for these divergent observations.

### ST6GalNAc6

ST6GalNAc6 catalyzes the synthesis of α-series gangliosides, including GD1α, GT1aα and GQ1bα (191), globo-series glycosphingolipids (GSL) (192, 193), and disialyl LeA (194, 195). In humans, ST6GalNAc6 is widely expressed in different organs (193). In human colon cancer ST6GalNAc6 is downregulated compared to nonmalignant epithelium, which is paralleled by a decrease in disialyl LeA expression and a concomitant increase in sialyl LeA (195). Such downregulation of ST6GalNAc6 occurs already in early-stage colon cancer and has been associated with epigenetic silencing (196). The related glycan change from disialyl LeA to sialyl LeA may increase E-selectin binding activity during metastasis and support inflammation-driven carcinogenesis by reduced binding to immunoregulatory Siglec-7 (195). mRNA levels of ST6GalNAc6 have also been found to be reduced in human kidney tumor lesions as compared to healthy tissue from the same patient (193). However, ST6GalNAc6 may also enhance the metastatic capability of tumor cells, as silencing of ST6GalNAc6 in a renal cell carcinoma (RCC) cell line, expressing lower levels of DSGb5, exhibited decreased migration, but not proliferation, *in vitro* (192). Siglec-7 binds to the RCC cell line ACHN in a DSGb5-dependent fashion and silencing of ST6GalNAc6 led to reduced surface binding of a Siglec-Fc chimera protein in these cells (197). These ST6GalNAc6 knockdown cells were more susceptible to cytotoxicity mediated by sialidase-treated NK cells *in vitro*, suggesting that this sialyltransferase has the potential to generate glyco-immune checkpoints at least in some types of tumors.

## ST8Sia Family

The ST8Sia family catalyzes the transfer of sialic acid to another sialic acid in an α2,8-linkage (60). Oligosialic acid chains display a chain of 2-7 sialic acids, whereas polysialic acid (polySia) chain exhibit a chain of eight or more polysialic acids (198). ST8Sia2 and 4 are also called polysialyltransferases as they participate in extending linear chains of polysialic acids (60). ST8Sia3 also participate in polysialylation, but with less efficacy than ST8Sia2 and 4 (199). ST8Sia1 (GD3 synthase), ST8Sia3, ST8Sia5 and ST8Sia6 are involved in the synthesis of sialylated glycolipids (60).

### ST8Sia1/GD3 Synthase

ST8Sia1 is also known as GD3 synthase (GD3S), as it catalyzes the transfer of a sialic acid residue onto GM3 to give raise to the b-series ganglioside GD3, which can eventually be further processed for the biosynthesis of other b-/c-series gangliosides (59). GD3S expression positively correlates with increasing grades of astrocytomas and is highly expressed in glioblastoma (200). In metastatic melanoma high ST8Sia1 expression is associated with detrimental outcome and higher expression in metastatic lesions, particularly in the brain (201). Recent studies analyzing data from The Cancer Genome Atlas (TCGA) showed an association of high ST8Sia1 expression levels in breast cancer with poor patient survival (202–204), which is eventually linked to epigenetic hypomethylation of the *ST8SIA1* gene (204). As opposed, in another study higher expression of ST8Sia1 mRNA in estrogen receptor (ER) positive breast cancer patients has been associated with higher disease free survival, while no significant difference was found in ER negative patients (205). However, a growing body of evidence supports the notion that ST8Sia1 is associated with tumor growth and progression. In a murine model of glioma, ST8Sia1-deficient mice exhibited attenuated glioma progression, lower-grade pathology and prolonged lifespan (206). Furthermore, in a breast cancer xenograft model silencing of ST8Sia1 led to reduced tumor growth and triptolide-mediated downregulation of ST8Sia1 inhibited tumor growth and prolonged survival (207). ST8Sia1 overexpression has been shown to bypass the need of serum for cell growth and to enhance migratory properties of breast cancer and glioma cell lines (208, 209). Inhibition of ST8Sia1 function by shRNA or triptolide affected the initiation and maintenance of EMT and ST8Sia1 expression correlated with activation of the c-Met signaling pathway enhancing stemness and metastatic properties (203). The implication of ST8Sia1 in stemness with c-Met signaling downstream of this enzyme was also found in experimental models of glioblastoma (200). ST8Sia1 activity has also been linked to oncogenic signaling through Wnt/b-catenin or Akt, Erk, and Src kinases (206, 210), which eventually may confer chemoresistance (210). GD3 has been identified as a ligand for Siglec-7 (81, 82), and ST8Sia1-transfected P815 cells with high surface expression of GD3 exhibited resistance to NK cell-mediated cytotoxicity due to Siglec-7-dependent inhibition (211).

### ST8Sia2/STX

The polysialyltransferase ST8Sia2, also known as sialyltransferase X (STX) is involved in the synthesis of linear polymers of sialic acid, so-called polysialic acid (polySia) chains (212). Polysialic acids are a form of post-translational modifications on different proteins, including the neural cell adhesion molecule (NCAM). Besides expression in healthy neuronal tissues, ST8Sia2 is expressed in neuronal and non-neuronal tumors and expression levels eventually correlate with advanced stage of disease, poor prognosis and risk of relapse (213–215). In an *in vivo* model, ST8Sia2-transfected glioma cells with high expression of polySia exhibited increased tumor invasion within the brain of recipient mice (216). Overexpression of *ST8SIA2* appears to also enhance invasiveness and metastatic capabilities of small cell lung cancer cells *in vitro* (217). Cytidine monophosphate (CMP) was reported to competitively inhibit ST8Sia2 and treatment with CMP led to reduced migration of ST8Sia2-expressing but not non-expressing cell lines in 2D migration assays (218). ST8Sia2 was upregulated in a subset of primary human carcinoma-associated fibroblasts (CAFs), and ST8SIA2 silencing in co-cultured CAFs resulted in decreased lung tumor cells invasion in a 3D model (215).

### ST8Sia3

ST8Sia3 is highly expressed in brain and testis and mediates the sialylation of a diversity of glycolipids (GM3, GD3 and α2,3-sialylparagloboside) and select glycoproteins, including striatal glycoproteins (199, 219, 220). ST8Sia3 can also transfer polySia to NCAM, but with a lower efficacy than ST8Sia2 and ST8Sia4 (199). ST8Sia3 was shown to promote survival, proliferation, clonogenicity, and migration of glioblastoma cells based on *ST8SIA3* knockdown experiments *in vitro* (221). Moreover, in the same study it was observed that mice xenografted intracranially with human glioblastoma cell line silenced for ST8Sia3 showed a better overall survival and tumors obtained from these mice demonstrated a lower Ki67 proliferation index.

### ST8Sia4/PST

ST8Sia4, also known as polysialyltransferase (PST), synthesizes slightly longer polySia chains compared to ST8Sia2, eventually conferring different molecular properties (222). Both polysialyltransferases are thought to contribute to the polysialylation of NCAM in mammalian cells (223). ST8Sia4 was also reported to be overexpressed in human RCC and breast cancer tissues and to promote cancer progression (224, 225). In these studies, silencing of ST8Sia4 by short-hairpin RNA (shRNA) or specific microRNA (miRNA) reduced cancer cell proliferation and invasion *in vitro*, and decreased tumor growth *in vivo*. High levels of ST8Sia4 expression was observed in chemoresistant leukemic cells (226–228), which may functionally contribute to chemoresistance, eventually by processes involving PI3K/AKT signaling (226, 227). However, in FTC patient tissues, *ST8SIA4* was observed to be downregulated compared to normal thyroid tissue, and ST8Sia4 expression in cell lines inversely correlated with proliferation, migration and invasion *in vitro* or tumor growth *in vivo* (229). Specific miRNAs targeting ST8SIA4 were reported to promote proliferation and invasion capabilities of FTC and oral squamous carcinoma cells (229, 230), and to foster epithelial-to-mesenchymal transition (230).

### ST8Sia5

ST8Sia5 exhibits transferase activity of sialic acid moieties onto several gangliosides to synthesize GT3, GD1c, GT1a and GQ1b, respectively (231, 232). Decreased expression of ST8SIA5 from TCGA dataset was linked to a poor survival in patients suffering from colon cancer, and decreased *ST8SIA5* transcript was also observed in a murine model of colitis-associated cancer (233). The reduced expression of ST8Sia5 was linked to gene regulation by forkhead box O3 (FOXO3), the functional deficiency of which may facilitate inflammation-mediated colon cancer growth (233).

### ST8Sia6

ST8Sia6 generates disialic acid structures, eventually by transfer of a sialic acid moiety onto a NeuAcα2,3 (6)Gal disaccharide on acceptor substrates, which include glycolipids, but preferentially O-linked glycoproteins (234). Some investigators suggest that ST8SIA6 Antisense RNA 1 (ST8SIA6-AS1) is associated with poor prognosis and enhances the proliferative and metastatic potential of cancer cells (235–240). Furthermore, ST8Sia6 may increase the chemosensitivity of tumor cells at least to certain drugs (226). However, *ST8SIA6* expression was found to be upregulated in several types of cancer and to be associated with a poor prognosis (241). Engineered murine colon and melanoma cancer cell lines expressing ST8Sia6 grew faster and led to a decreased survival *in vivo* and depending on host Siglec-E (241). Also depending on Siglec-E, *ST8SIA6* expression induced an antitumor immune responses characterized by macrophage polarization toward M2 and upregulation of arginase, which required Siglec-E (241). Notably, 2,8-disialic acid structures were shown to be ligands of murine Siglec-E (242), as well as human Siglec-7 and -9 (81, 241), and may thus act as glyco-immune checkpoints in human cancer.

## Sialic Acid-Binding Proteins in Cancer

Sialyltransferases are involved in the biosynthesis of tumor-associated sialoglycans, which *via* recognition by sialic acid-binding proteins, influence tumor progression and the immune response of the host. Siglecs and selectins are among the most intensively studied sialic acid-binding lectins, and their implication in cancer will be briefly discussed in this section.

### Siglecs

Sialic acid-binding immunoglobulin-type lectins (Siglecs), are a family of I-type lectins that belong to the immunoglobulin superfamily. Siglecs are cell-surface receptors predominantly expressed on leukocytes in a cell-specific and differentiation-dependent manner (243). On the basis of evolutionary conservation and sequence similarity, they are divided into two subsets: the first comprises sialoadhesin (also known as Siglec-1 and CD169), CD22 (also known as Siglec-2), myelin-associated glycoprotein (MAG; also known as Siglec-4) and Siglec-15 (244), and are quite distantly related (∼25-30% sequence identity) (245). The other group comprises CD33-related Siglecs (Siglec-3 (CD33), Siglec-5, Siglec-6, Siglec-7, Siglec-8, Siglec-9, Siglec-10, Siglec-11, Siglec-14, and Siglec-16), which have ∼50-99% identity and have evolutionary rapidly evolved due to exon shuffling, exon loss, gene conversion and gene duplication (244, 245). Structurally, Siglecs consist of an amino-terminal V-set domain that confers binding specificity for select sialoglycan ligands, which differ across individual family members (246), and between species (245). The V-set domain is followed by a differing number of immunoglobulin-like domains, a transmembrane domain, and the carboxy-terminal cytoplasmic tail that contains inhibitory, or for fewer members activating, signaling motifs (247). It has been proposed that Siglec ligands might serve as self-associated molecular patterns (SAMPs) to avoid autoreactivity of immune cells (248).

Ligands for Siglecs are broadly expressed in different types of human tumors and in a diversity of common cancer cell lines (18). The expression of Siglec-7 and -9 ligands protected tumor cells from NK cell-mediated cytotoxicity *in vitro*, and in a Siglec humanized *in vivo* model (18). In a complementary approach, it was shown that tumor cells decorated with synthetic glycopolymers inhibited NK cell cytotoxicity by engagement of Siglec-7 (249). The body of evidence for Siglec-mediated immune checkpoints in cancer is rapidly growing and indicates that the sialic acid-Siglec axis is relevant for the control of both myeloid and lymphoid immune cells within the tumor microenvironment (12, 16, 17). Interestingly, Siglecs have been shown to be up-regulated on subsets of tumor-infiltrating and circulating cytotoxic T cells in cancer patients (20, 250), in particular on functionally potent effector memory and EMRA T cells (20). While a variety of Siglec-based therapeutic strategies for cancer immunotherapy are currently under investigation (17, 251), a better understanding of the identity and expression not only of tumor-associated sialoside ligands, but also of underlying carrier molecule (252, 253), in specific tumors and patients, may allow for more tailored treatment strategies.

### Selectins

Selectins are a family of three calcium-dependent (C-type) lectins comprising E-selectin, L-selectin, and P-selectin, named after their expression on endothelial cells, leukocytes and platelets. In contrast to L-selectin that is constitutively expressed on leukocytes and E-selectin in postcapillary venules of the skin and bone marrow (17), however, E- and P-selectin expression on endothelial cells or platelets are mainly induced following cellular activation (254). The main physiological function of all selectins is to mediate the rolling and adhesion of leukocyte during leukocyte recruitment to sites of inflammation or to lymphoid tissues (254). The carbohydrate-recognition domain (CDR) of all selectins has modest affinity to sLeX and its isomer sLeA (254), which are among the best described ligands for selectins (17). The synthesis of these tetrasaccharides occurs due to the integrated action of α2,3-sialyltransferases with α1,3-fucosyltransferases, β1,4-galactosyltranferases, and *N*-acetyl-β-glucosaminyltransferases (255). As discussed above, ST3Gal3, ST3Gal4 and ST3Gal6 are involved in the synthesis of sLeX, while sLeA is predominantly generated by ST3Gal3.

sLeA and sLeX are known tumor markers and functionally implicated in the malignant behavior of cancer cells (88). Glycosylated proteins carrying sLeX/A moieties, such as PSGL-1, CD24, CD44, ESL-1, and death receptor-3 represent major selectin ligands on cancer cells (14). The overexpression of selectin ligands has been linked to cancer progression and poor prognosis in some cancers (14, 88, 256). *In vivo* studies using selectin knockout or selectin ligand deficient mice highlighted the importance of selectins in metastasis (3). Selectins seems to contribute to metastasis through heterotypic interactions between tumor cells, leukocytes and endothelial cells (14, 256). These interactions may also foster tumor embolus formation with local activation of endothelial cells and increased transendothelial migration of both tumor cells and leukocytes (3). Recruited leukocytes might further enhance vascular permeability and cancer cell extravasation, and also shape the tumor microenvironment (14). While earlier studies on selectin-targeted therapies focused on cardiovascular disease, positive outcomes from clinical trials have raised the interest in strategies targeting selectin receptor-ligand interactions in cancer.

## Conclusion

In the last decade we have witnessed a significant body of discoveries that highlight the importance of sialic acids in cancer biology and immuno-oncology. As biosynthetic enzymes for sialosides, human SiaTs have long been linked to cancer hypersialylation. However, the twenty SiaTs exhibit different characteristics and their roles in cancer are manyfold and complex, and remain to be fully explored. The expression of SiaTs, sialosides and sialic acid interaction partners (e.g. Siglecs), can vary between different types of tumors, between primary tumor and metastatic lesion, and even between patients (19). Furthermore, controversial observations on the role of a select SiaT may be due to its involvement in the synthesis of multiple glycans, eventually generating various ligands for different glycan-binding proteins. Moreover, limitations of methodological approaches need to be considered, such as missing environmental context for *in vitro* cell cultures or species differences for *in vivo* studies. Functional redundancy may exist between SiaTs, and while specific small-molecule SiaT inhibitors that bind and block select SiaTs may hold promise for therapeutic and diagnostic use [for recent reviews see (257–259)], combination strategies might be needed in a given context. However, the observation that SiaTs are responsible for the generation of glyco-immune checkpoints has reinvigorated ambitions of researchers to explore the role of individual SiaTs in cancer, which may pave the way for novel immune normalization (260), and more personalized, cancer immunotherapies.

## Author Contributions

All authors wrote and approved the manuscript.

## Funding

The laboratory of SV is supported by grants from the Swiss National Science Foundation (310030_184757), the Swiss Cancer League/Swiss Cancer Research (KFS-4958-02-2020), and the Bern Center for Precision Medicine (BCPM).

## Conflict of Interest

SVG received remuneration for serving on the scientific advisory board of Palleon Pharmaceuticals.

The remaining authors declare that the research was conducted in the absence of any commercial or financial relationships that could be construed as a potential conflict of interest.

## Publisher’s Note

All claims expressed in this article are solely those of the authors and do not necessarily represent those of their affiliated organizations, or those of the publisher, the editors and the reviewers. Any product that may be evaluated in this article, or claim that may be made by its manufacturer, is not guaranteed or endorsed by the publisher.
